# Memory-based meso-scale modeling of Covid-19: County-resolved timelines in Germany

**DOI:** 10.1007/s00466-020-01883-5

**Published:** 2020-08-03

**Authors:** Andreas Kergaßner, Christian Burkhardt, Dorothee Lippold, Matthias Kergaßner, Lukas Pflug, Dominik Budday, Paul Steinmann, Silvia Budday

**Affiliations:** 1 Department of Mechanical Engineering, Institute of Applied Mechanics, Friedrich-Alexander-University Erlangen Nürnberg, 91058 Erlangen, Germany; 2 Department of Computer Science, Hardware-Software-Co-Design, Friedrich-Alexander-University Erlangen-Nürnberg, 91058 Erlangen, Germany; 3 Department of Mathematics, Chair of Applied Mathematics (Continuous Optimization), Friedrich-Alexander-University Erlangen-Nürnberg, 91058 Erlangen, Germany; 4 Central Institute for Scientic Computing (ZISC), Friedrich-Alexander-University Erlangen-Nürnberg, Martensstrasse 5a, 91058 Erlangen, Germany

**Keywords:** COVID-19, SIR and integro-differential models, Spatio-temporal meso-scale outbreak dynamics

## Abstract

The COVID-19 pandemic has led to an unprecedented world-wide effort to gather data, model, and understand the viral spread. Entire societies and economies are desperate to recover and get back to normality. However, to this end accurate models are of essence that capture both the viral spread and the courses of disease in space and time at reasonable resolution. Here, we combine a spatially resolved county-level infection model for Germany with a memory-based integro-differential approach capable of directly including medical data on the course of disease, which is not possible when using traditional SIR-type models. We calibrate our model with data on cumulative detected infections and deaths from the Robert-Koch Institute and demonstrate how the model can be used to obtain county- or even city-level estimates on the number of new infections, hospitality rates and demands on intensive care units. We believe that the present work may help guide decision makers to locally fine-tune their expedient response to potential new outbreaks in the near future.

## Introduction

The COVID-19 pandemic continues to hold our way of life on this planet in a tight grip. Over the whole world, we have now reached more than 10 million infections [1]. While new infections still rise at alarming pace in the United States, Brazil, or India, most other Asian and European countries that were hit much earlier by the pandemic seem to have succeeded in reducing the number of new daily cases. This success can largely be attributed to fast and locally tailored political measures that introduced severe travel restrictions [2–6] and curtailed public life. Initially, the measures were met by a largely understanding general public. However, the partial necessity of police enforcement and increasing protest against contact restrictions, locally even encouraged by politicians [7], demonstrate rising anger, fear, or even mental health problems due to the current situation [8, 9]. Thus, it is critical to carefully reopen the economy and reestablish public life, while avoiding a relapse and a potential collapse of the health-care system, which may entail much stricter measures again. To reach this goal, however, decisions must be made quickly and often locally at county level, based on reliable data and trustworthy predictions. Clearly, accurate models are of essence to capture the disease dynamics at exactly this spatial meso-scale, to predict the number of new infections per day or the number of patients that may require intensive care.

Here, we focus on the situation in Germany, where county-resolved daily and cumulative infection cases are reliably reported by the Robert-Koch Institute (RKI, [10]). We combine two previous modeling advances [11, 12] into a locally resolved, history-type model that captures the spatio-temporal evolution of the pandemic in Germany. We use a generalization of typically known SIR-type compartment models that allows for a much better representation of the courses of disease [12]. While becoming infected is well represented by a simple ordinary differential equation (ODE), the remaining course of disease is captured rather restrictively by these ODE-based, SIR-type models [13].

Based on the integro-differential model introduced by Kermack and McKendrick already in 1927 [14] and recently reintroduced by [12, 13], we model the spatial spread of Covid-19 in the following way:

$$\begin{aligned} \dot{\mathrm {S}}(t,x)&= \mathrm {S}(t,x) \int _{\varOmega } \beta (t, x, y) \int _{0}^\infty \gamma _I(\tau )\dot{\mathrm {S}} (t-\tau ,y) \, \mathrm {d} \tau \, \mathrm {d}y, \\ \mathrm {S}(s,x)&= \mathrm {S}_0(s,x), \end{aligned}$$

for all $s < 0, t > 0$ and $x\in \varOmega$ with $\varOmega \subset \mathbb {R}^2$ open denoting the considered spatial region. The normalized initial history datum is given by $S_0\in W^{1,\infty }((-\infty ,0);L^\infty (\varOmega ;[0,1]))$. The function $S \in W^{1,\infty }((0,\infty );L^\infty (\varOmega ;[0,1]))$ denotes the normalized number of susceptibles. The weight $\gamma _I \in L^{1}((0,\infty ) ; \mathbb R_{\ge 0} ))$ with $\Vert \gamma _I\Vert _{L^1((0,\infty ))}=1$ describes the evolution of infectiousness, where $\gamma _I(\tau )$ defines the infectiousness of an individual at $\tau$ days after the infection event. The interaction term $\beta \in L ^\infty ((0,\infty )\times \varOmega ^2;\mathbb {R}_{\ge 0})$ denotes the interaction between the infectious and the susceptible population.

The considered balance law is of nonlocal-history type. Nonlocality as well as history in balance laws are receiving increasing attention to model real world phenomena. They provide a more detailed way to model evolution and can be seen as the mesoscopic link between purely macroscopic and fully microscopic models. In the considered application, the microscopic equivalent—agent models [15–17]—can be interpreted as a measure valued solution to the proposed model.

The classically used compartment models (SIR, SEIR) [18] have been widely used to model the viral spread. Their recently revealed relationship to Hamiltonian mechanics is quite insightful [19], demonstrating that they constitute a mere simplification of the here considered integro-differential equations. In terms of the spatial resolution—which of course can also be modeled in the compartment models [20, 21]—the classical SIR model can be seen as the singular limit of the interaction term, i.e. $\beta (\cdot ,*,\star ) \rightarrow b(\cdot ) \delta (*-\star )$ for a given $b \in L ^\infty ((0,\infty );\mathbb {R}_{\ge 0})$. The models are generalized with respect to the evolution of infectiousness of infected individuals. The considered model can represent—based on medical data—any course of infectiousness, in contrast to, e.g., an assumed exponential decay in the widely used SIR model.

As introduced in [11], we discretize our spatial domain $\varOmega$, Germany, at county-level (or even city-level), where current containment rules are steadily evaluated and adapted in case local infection numbers rise up again. Our county-interaction network is adapted from the GLobal Epidemic And Mobility (GLEAM) model [22, 23], focusing on mid- and short-range interactions motivated by the severely restricted air travel [24, 25]. Taken together, this spatially resolved integro-differential model allows us to accurately analyze and predict disease dynamics at its various stages and the effect of local measures.

## Governing equations

### Memory-based spatial infection model

To model the spread of the disease in a discretized spatial setting, we consider a finite partition of the domain $\varOmega$, i.e. $(\varOmega _k)_{k\in \{1,\ldots ,N\}}$ with $\varOmega _k \subset \varOmega$ and $\cup _{k=1}^N \bar{\varOmega }_k = \bar{\varOmega }$, where *N* denotes the number of counties or cities in Germany, depending on the spatial resolution. We obtain the following memory type vector-valued initial value problem

1 $$\begin{aligned} \dot{\mathbf {S}}(t)&= \overline{\mathbf {S}}(t) ~ \mathbf {B}(t) \int _{0}^\infty \gamma _I(\tau )\dot{\mathbf {S}} (t-\tau ) \, \mathrm {d} \tau&\forall t > 0, \\ \mathbf {S}(t)&= \mathbf {S}_0(t)&\forall t < 0, \nonumber \end{aligned}$$

where $\mathbf {S}_0 \in W^{1,\infty }((-\infty ,0);[0,1]^N)$ is the vector-valued normalized initial history datum. We introduce $\overline{\bullet }$ to denote the transformation of a vector $\bullet$ into a quadratic diagonal matrix, where the entries along the diagonal equal those of the vector. The time-dependent, vector-valued function $\mathbf {S}\in W^{1,\infty }((0,\infty );[0,1]^N)$ denotes the normalized number of susceptibles. The matrix-valued function $\mathbf {B}\in L^{\infty }((0,\infty ); \mathbb {R}^{N\times N}_{\ge 0})$ denotes the infection rates and interaction between the considered regions $\varOmega _k$ with $k\in \{1,\ldots ,N\}$. The existence and uniqueness of a solution of the proposed integro-differential equations—continuous as well as discretized in space—is proven e.g. in [12] for all $\gamma _I$ for which there exists $\epsilon > 0$ s.t. $\gamma _I|_{(0,\epsilon )} \equiv 0$. This is a rather natural condition, since the incubation time—the period during which the infected are not yet infectious—is positive.

**Fig. 1:**
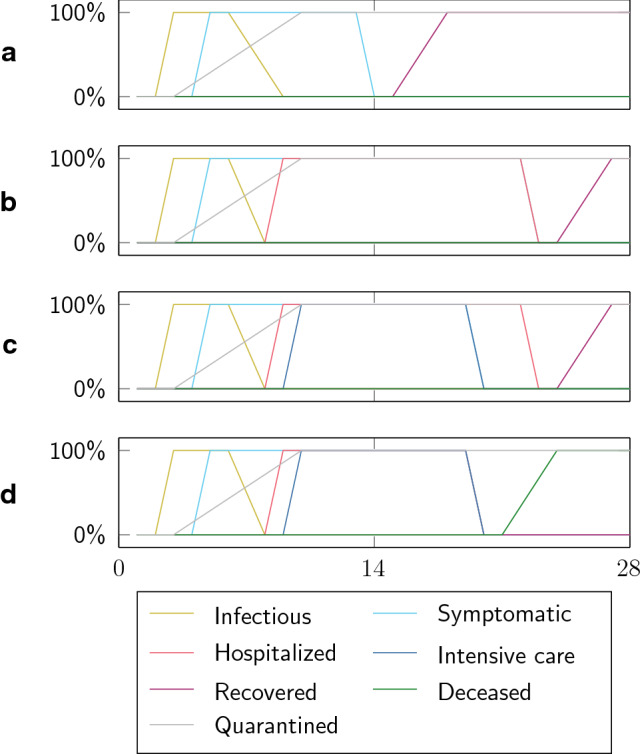
Four different courses of Covid-19 disease with a total duration of 28 days: **a** light symptoms, recovering without hospitalization; **b** hospitalization, recovering without intensive care; **c** patients in intensive care and recovering; **d** patients in intensive care that eventually die

Based on the history of $\mathbf {S}$, other quantities and subgroups can be determined directly from including medical data on the various courses and infectiousness levels of the disease via corresponding integration weights: We distinguish between the states infectious $\gamma _I$, symptomatic $\gamma _S$, tested and quarantined $\gamma _Q$, hospitalized $\gamma _H$, in intensive care $\gamma _{\text {ICU}}$, recovered $\gamma _R$ and deceased $\gamma _{D}$. Following the contribution of [26], we include four different courses of Covid-19 disease progression in our model: (a) light symptoms, recovering without hospitalization, 95% share; (b) hospitalization, recovering without intensive care, 4% share; (c) patients in intensive care and recovering, 0.4% share; (d) patients in intensive care that eventually die, 0.6% share. Figure 1 depicts the four different courses of the disease as represented in our model, including their corresponding state transitions and infectiousness levels. Note that only the weighted sum $\gamma _I = \sum _{i=1}^{4} \text {share}_i \cdot \gamma _{I,i}$ is necessary for the solution of Eq. (1), but the individual contributions allow for detailed descriptions of disease progression from medical data and corresponding post processing. We normalize the integral over $\gamma _I$ such that it represents the probability of infection.

**Fig. 2:**
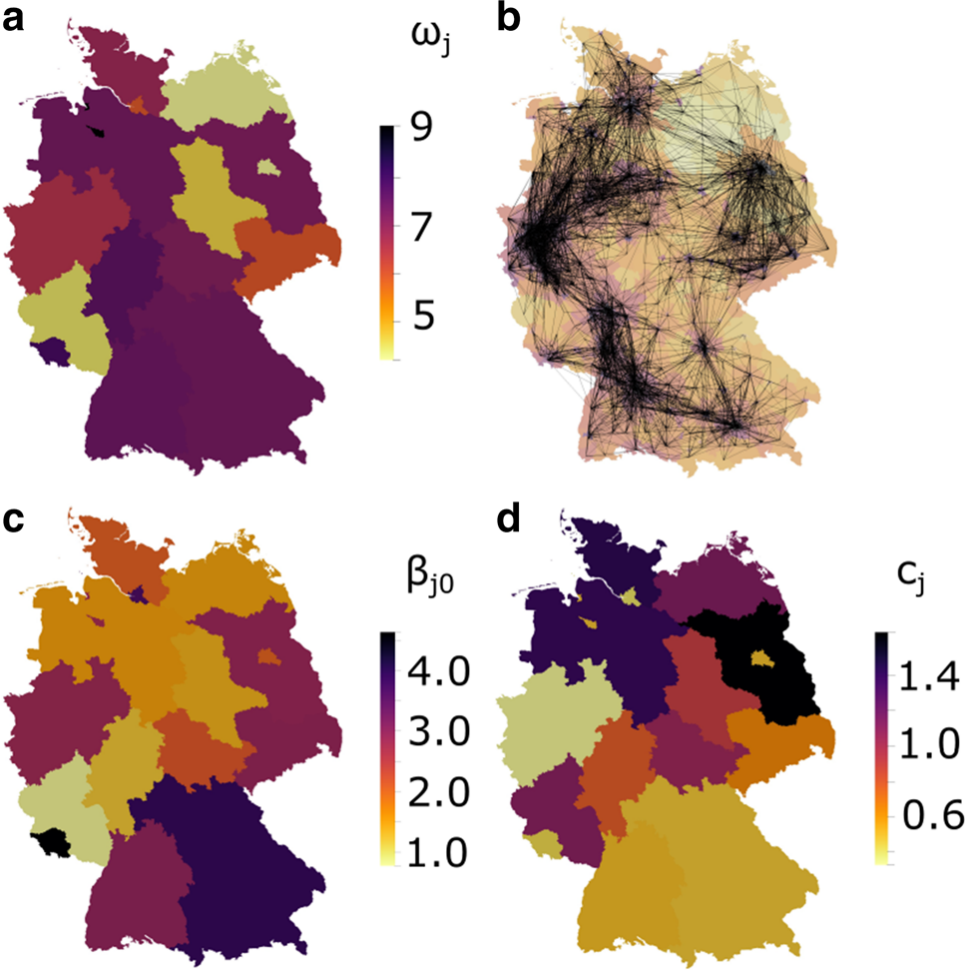
Model parameters and cross-county interactions. **a** State-wise estimated dark ratios $\omega _j$ following from an assumed constant mortality across Germany. **b** Major connectors (lines) between counties of the network mobility model to predict cross-county infections. County color varies from yellow to dark purple with population density. Illustration of state-wise optimized values for $\beta _{j0}$ (**c**) and $c_j$ (**d**) with $j=\{1,\ldots , 16\}$

We further assume that patients in courses (b) to (d) will be tested positive and are thereby considered in reported infection numbers. The ratio of total versus detected infections is defined as dark ratio $\omega$, thereby representing the factor of unknown cases. Since the dark ratio is not necessarily constant in space, this is taken into account by locally scaling the function $\gamma _Q$ that represents the detected and quarantined state of course (a) to the appropriate value. Since the dark ratio seems to closely correlate with testing capacities [11], we introduce federal-state-wise dark ratios $\omega _j$, assembled in the vector $\varvec{\omega }$, that vary only over states with $j\in \{1,\ldots ,16\}$, but are identical for each county within one state (see Sect. 2.3 and Fig. 2a).

Due to locally differing behavior patterns and, in particular, political measures to reduce social contacts, the infection rates vary in space and time. Based on our previous findings in [11], we introduce federal-state-wise initial infection rates $\beta _{j0}$ with $j\in \{1,\ldots ,16\}$, and two reduction factors $\beta ^\text {red}_{1}$ and $\beta ^\text {red}_{2}$ representing the major restrictions of 1) cancelling large events and 2) contact restrictions. Those model parameters are calibrated using data reported by the RKI, as described in Sect. 2.3. Since the shut-down measures were introduced at slightly different times $T_{j1}$ and $T_{j2}$ in the different federal states of Germany, we model the time-dependent reduction of infection rates via piece-wise constant functions $\hat{\beta }_{j1} (t)$ and $\hat{\beta }_{j2} (t)$ to obtain the overall infection rates in each state *j* for $t\in \mathbb {R}_{\ge 0}$

2 $$\begin{aligned} \beta _j (t) = \beta _{j0} \hat{\beta }_{j1}(t) \hat{\beta }_{j2}(t), \ \hat{\beta }_{ji}(t) = {\left\{ \begin{array}{ll} 1, &{} \text {if} \; t < T_{ji}, \\ \beta ^\text {red}_{i} &{} \text {else.} \end{array}\right. } \end{aligned}$$

To model cross-county interactions, we adapt the GLEAM short- and mid-range mobility network [22, 23] as introduced in [11] and capture cross-county infections by

3 $$\begin{aligned} \begin{aligned} B_{_{kl}}(t)&= {\left\{ \begin{array}{ll} \beta _{_{k}} (t)&{}\text {if k=l} \\ c_k \sqrt{\beta _{_{k}}(t) \beta _{_{l}}(t)} \frac{N_k^\kappa N_l^\lambda }{N_{\text {max}}^{\kappa + \lambda }} \exp \left( - \frac{r_{_{kl}}}{r} \right) &{}\text {else} \end{array}\right. } \end{aligned} \end{aligned}$$

where $\beta _k$ are time-dependent infection rates, $c_k$ are the cross-county infection weights, $N_{k}$ is the number of inhabitants in the largest city of county *k*, $N_{max}=3e6$ corresponds to the number of inhabitants in Germany’s largest city Berlin, and $r_{kl}$ is the distance between counties *k* and *l*. Importantly, $\beta _k$ and $c_k$ are identical for all counties within one federal state, such that Eq. 3 introduces 16 additional model parameters $c_j$ with $j\in \{1,\ldots ,16\}$ which need to be calibrated based on reported data in the literature (see Sect. 2.3). The parameters for the GLEAM model are taken from [22] and given in Table 1. Figure 2b displays the mobility network across Germany [11]. Note that both the county-internal as well as the cross-county infections contribute to the basic reproduction number in our spatial model.

**Table 1:** Mobility network parameters as derived in [22]

$r_{kl}$ (km)	$\kappa$	$\lambda$	*r* (km)
$\le 300$	0.46	0.64	82
$>300$	0.35	0.37	1

For the distinction of individual counties, we use the municipal directory (*Gemeindeverzeichnis*) from the German Federal Statistical Office (*Statistisches Bundesamt*) [27], which delivers area, shape and population data. We consider city-wise population data, which is accumulated over the entire county or corresponding spatial domain for the respective model. The center of population of each county serves as its spatial coordinates.

The detailed description of the courses of disease allows for elaborate post-processing of the solution to evaluate any described quantity. Generally, evaluation differs for cumulative and current quantities. The number of cumulative discovered infections $\mathbf {Q}$ or the number of deceased $\mathbf {D}$ are evaluated by double integrals such as

4 $$\begin{aligned} \mathbf {Q}(t) = \int \limits _{-\infty }^t \int \limits _{0}^{\infty } \dot{\mathbf {S}}(t-\tau ) \, \gamma _Q(\tau ) \, \mathrm {d}\tau \, \mathrm {d}t \,. \end{aligned}$$

Current quantities like infectious $\mathbf {I}(t)$, those with symptoms, hospitalized or ICU patients follow from expressions such as

5 $$\begin{aligned} \mathbf {I}(t) = \int \limits _0^\infty \dot{\mathbf {S}}(t-\tau )\gamma _I(\tau ) \mathrm {d}t \, . \end{aligned}$$

### Initial conditions

From data the number of positively tested people on day zero is known, but the integro-differential equation model requires initial values for the infected at each spatial node as well as an initial history as a starting point for the integration. Initially, we assume exponential growth in all counties described by the ansatz

6 $$\begin{aligned} \mathbf {S}(t) = \mathbf {1} - \varvec{\varepsilon } \, \text {exp}(\nu t) \end{aligned}$$

with $\nu = 0.345$ from fitting an exponential function to RKI data on cumulative COVID-19 cases in all of Germany from March 2 to March 6. The initial estimated number of infected $\varvec{\varepsilon }$ at time $t=0$ in each county can be calculated by combining Eq. (4), the number of initially reported cases $\mathbf {Q}_0$, Eq. (6) and the time derivative $\dot{\mathbf {S}}$. From the result

7 $$\begin{aligned} \varvec{\varepsilon }^{-1} = \overline{\mathbf {Q}}{}_0^{-1} \int \limits _0^{\infty } \mathrm {exp}(-\nu \tau ) \, \gamma _Q(\tau ) \, \mathrm {d}\tau \end{aligned}$$

and Eq. (6), the initial history can be estimated.

The high-resolution network model brings with it the challenge for spatially consistent initial conditions. However, most counties did not yet have any known cases on the very first day, limiting the possibility of simply scaling overall initial infections per county by the dark ratio. Thus, for the county-based model we selected the distribution of initial infections according to data for March 16 [10], linearly scaling down the overall number of infections to the number reported on our starting date March 2.

### Fitting procedure

To approach our spatially resolved county-model, we followed a cascade optimization strategy. Data analysis and preliminary simulations had shown that we require federal-state-dependent dark ratios $\omega _j$ and infection rates $\beta _{j0}$, $j \in \{1,\ldots ,16\}$ [11]. Figure 2a illustrates the estimates for the state-wise dark ratios $\omega _j$, which we obtain by assuming a Germany-wide identical mortality of $\mu =6$‰[26] and fitting to the individually reported death tolls, with $\langle \varvec{\omega }\rangle \approx 6.5$. Using state-wise identified dark ratios $\omega _j$, we first used a coupled system of 16 nodes connecting each federal state to obtain a Germany-wide average $\beta$ and reduction factors $\beta ^{\text {red}}_1$ and $\beta ^\text {red}_{2}$ by fitting the cumulative data for Germany via the following objective function

8 $$\begin{aligned} \begin{aligned} {R_1}^2&= \int _{t_{\text {start}}}^{t_{\text {end}}} w_I\left[ \mathbf {Q}_{\text {RKI}}(t) - \mathbf {Q}(t) \right] ^2 \mathrm {d}t\\&\quad + w_D \left[ \mathbf {D}_\text {RKI}(t_{\text {end}}) - \mathbf {D}(t_{\text {end}})\right] ^2\\&\quad + w_S \left[ \dot{\mathbf {Q}}_{\text {RKI}}(t_{\text {end}}) - \dot{\mathbf {Q}}(t_{\text {end}}) \right] ^2. \end{aligned} \end{aligned}$$

The residual $R_1$ is minimized using a particle swarm optimization (PSO) algorithm described in detail in Sect. 2.4 with weights $w_I = 1/(t_\text {end}-t_\text {start}) / \text {max}(\mathbf {Q}_\text {RKI})$, $w_D = 1/\text {max}(\mathbf {D}_\text {RKI})$ and $w_S = 0.1/\text {max}(\dot{\mathbf {Q}}_\text {RKI})$.

We then considered state-wise data to fit $\beta _j, j \in \{1,\ldots ,16\}$, while keeping $c=1$, leading to the distribution over Germany depicted in Fig. 2c. We fit the cumulative number of confirmed infections reported by the RKI [10] for the time period from March 2 until April 25 with the cumulative number of detected infections $\mathbf {Q}$ as defined in Eq. (4), normalized by the maximum cumulative number of reported infections from the RKI. This is the time period during which the various shutdown measures were in place without any noticeable relaxation. On top of that, we include the change-rate of infections on our last day April 25 into the residual vector

9 $$\begin{aligned} \begin{aligned} {R_2}^2&= \int _{t_{\text {start}}}^{t_{\text {end}}} \sum _{j=1}^{16} w_{I,j} \left[ \mathbf {Q}_{\text {RKI}}(t) - \mathbf {Q}(t) \right] ^2 \mathrm {d}t\\&\quad + \sum _{j=1}^{16} w_{D,j} \left[ D_\text {RKI}(t_{\text {end}}) - D(t_{\text {end}})\right] ^2\\&\quad + \sum _{j=1}^{16} w_{S,j} \left[ \dot{Q}_\text {RKI}(t_{\text {end}}) - \dot{Q}(t_{\text {end}}) \right] ^2 \, . \end{aligned} \end{aligned}$$

with the weights

10 $$\begin{aligned} w_{I,j}&= \frac{1}{(t_\text {end}-t_\text {start})}\,/\, \frac{\text {max}(\mathbf {Q}_{\text {RKI},j})+\text {max}(\mathbf {Q}_{\text {RKI},j})}{2}\nonumber \\ w_{D,j}&= 1/~\frac{\text {max}(\mathbf {D}_{\text {RKI},j})+\text {max}(\mathbf {D}_{\text {RKI},j})}{2}\\ w_{S,j}&= 0.1/~\frac{\text {max}(\dot{\mathbf {Q}}_{\text {RKI},j})+\text {max}(\dot{\mathbf {Q}}_{\text {RKI},j})}{2}\nonumber \, . \end{aligned}$$

The weights are state-wise normalized to balance the contribution of heavily and less affected states. The residual $R_2$ is again minimized using the PSO algorithm. Finally, we increased the resolution to full county level, amounting to a coupled system of 401 nodes. To re-balance the changed influence of the larger network, we iteratively fitted state-wise cross-county weights $c_j$ for $\in \{1,\ldots ,16\}$ to match the state-wise cumulative infections of the 16 node state-wise model ($Q_{\text {sw}}$) with the accumulated numbers from the 401 node county-based model ($Q_{\text {cw}}$) on the last day of the fit. We used a damped gradient-descent like algorithm to update $c_j$ at iteration $i+1$ following the rule

11 $$\begin{aligned} \begin{aligned} c_j^{i+1} = c_j^{i} \, \mathrm {min}(\varDelta c_{\text {max}},\, \mathrm {sign}(R_3) r^{\zeta })\\ \text {with } \quad R_3 = \frac{Q_{\text {sw}}(t_{\text {end}}) - Q_{\text {cw}}(t_{\text {end}})}{Q_{\text {sw}}(t_{\text {end}})} \end{aligned} \end{aligned}$$

Empirically, we obtained converged cross-county weights within 30 iterations with a limited step size $\varDelta c_{\text {max}} = 0.25$ and a damping exponent $\zeta = 1.5$. The final state-wise distribution of optimized cross-county weights $c_j$ is displayed in Fig. 2d.

### Particle swarm optimization

Particle swarms are distributed optimization schemes that treat each realization of the *d* optimization variables as particles with a position $\mathbf{x} ^i$ and a velocity $\mathbf{v} ^i$ in a *d*-dimensional bounded search space. Particles are initialized with a uniformly random position within the boundaries of the search space and zero initial speed. For the iteration $i>0$ the following set of equations describes the behaviour of any particle:

12 $$\begin{aligned} \mathbf{v} ^{i+1}&=a\mathbf{v} ^i + b_\text {loc} \mathbf{R} _\text {loc}^i [\mathbf{p} _\text {loc}^i -\mathbf{x} ^i] + b_\text {glob}{} \mathbf{R} _\text {glob}^i[\mathbf{p} _\text {glob}^i-\mathbf{x} ^i], \nonumber \\ \mathbf{x} ^{i+1}&=\mathbf{x} ^i+\mathbf{v} ^{i+1}. \end{aligned}$$

The velocity $\mathbf{v} ^{i+1}$ is a linear combination of three quantities. The previous velocity $\mathbf{v} ^i$ weighted with the constant factor *a* results in an inert motion property. The term $\mathbf{p} _\text {loc}^i -\mathbf{x} ^i$ represents a force that pulls the particle towards its local attractor $\mathbf{p} _\text {loc}^i$, which is the best position this specific particle has visited so far. Multiplication with the constant weight $b_\text {loc}$ controls the influence of this quantity. In addition to that the randomized diagonal matrix $\mathbf{R} _\text {loc}^i$ with values between 0 and 1 enables optimization in varying directions. The global attractor $\mathbf{p} _\text {glob}^i$ represents the best position any particle has visited so far and works analogously to the local attractor with the factor $b_\text {glob}$.

**Table 2:** Parameters of the particle swarm [28]

*a*	0.72984
$b_\text {loc}$	1.496172
$b_\text {glob}$	1.496172

We chose established values for *a*, $b_\text {loc}$ and $b_\text {glob}$ as summarized in Table 2, used a total of 300 particles and followed the ‘nearest’ strategy when particles cross boundaries of the search space during optimization [29]. To prevent overly fast convergence to a visited attractor without broad coverage of the search space, we employed a so-called ring topology neighborhood, such that the global attractor of a particle corresponds only to the best local attractor of its two neighbors below and above. This way, good positions are slowly propagated through the whole swarm, allowing for enhanced exploration of the search space, which well balances run-time efficiency and identification of the true global optimum.

**Fig. 3:**
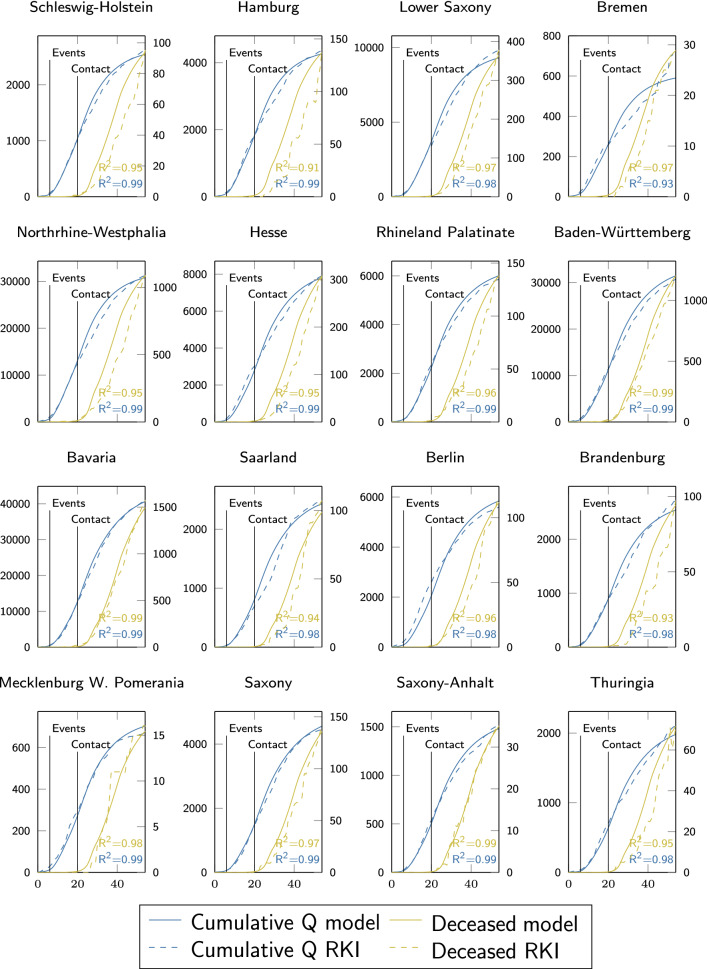
Model (solid line) calibration using the state-wise evolution of cumulative quarantined infections **Q** (left axis, blue) reported by the RKI (dashed) from March 2 to April 25 (*x* in days since March 2) and the dead count on April 25 (right axis, light green). Vertical lines denote changes in $\varvec{\beta }$. $R^2$ indicates Pearson-correlation of data and the simulated spread

### Statistical analysis

To validate the model, we evaluated the temporal correlation between model predictions and RKI data by computing the Pearson correlation coefficient $r_P$, the coefficient of determination $R^2 = r_P^2$ and the corresponding *p*-value to assess statistical significance via the function [$r_P$,*p*] = *corrcoef*(...) in *Octave* 5.2.0. Note that $r_P$ by definition only reports on the linear correlation and has to be taken with caution.

## Results and discussion

### Model calibration and validation

Figure 3 shows how the optimized spatially resolved memory-based model with 401 network nodes representing each county of Germany well reproduces the cumulative confirmed cases in each of its federal states from March 2 until April 25. For cumulative infection data reported by the RKI [10], we find astonishing and statistically significant (all $p < 1e-12$) agreement on the temporal evolution. The only state with an $R^2 < 0.98$ is Bremen—a city-state with overall very low infection numbers and a population of less than 700.000. Here our quasi-continuum modeling approach and the underlying exponential growth seem to approach their validity limit, and stochastic effects start to prevail. Although only the last data point of reported deaths was considered for parameter identification, the model captures the temporal evolution of Covid-19 related deaths in each state of Germany with remarkable accuracy (all $R^2 > 0.91$). Here, we observe least agreement in the city-state Hamburg. In general, the model better captures the evolution in higher-populated states, with overall more infections and death tolls.

**Fig. 4:**
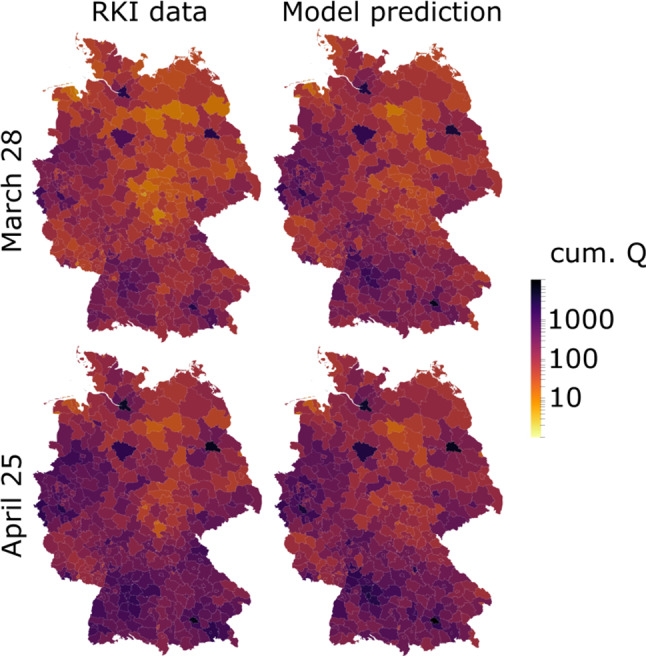
Spatial distribution of county-wise cumulative discovered infections $\mathbf {Q}$ on March 28 (top) and April 25 (bottom) as reported by the RKI (left) and simulated by our model (right), calibrated using federal-state-wise information only

**Fig. 5:**
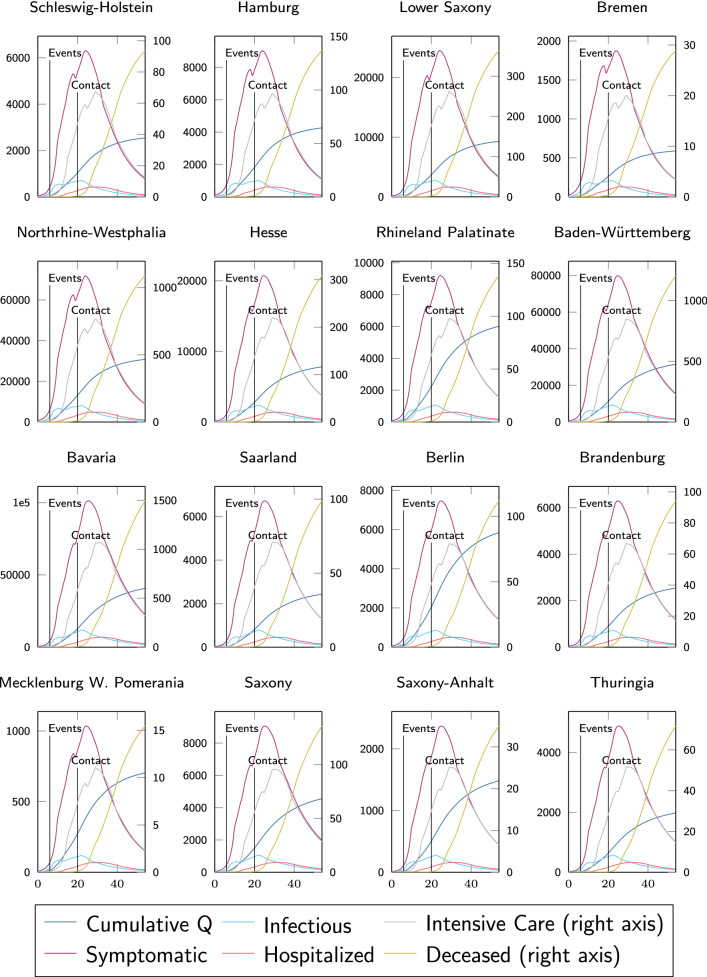
Simulated state-wise temporal evolution from March 2 until April 25 of cumulative quarantined (dark blue), symptomatic (purple), infectious (light blue), hospitalized (light red), patients in intensive care (gray), and dead (dark yellow) using the calibrated model

**Fig. 6:**
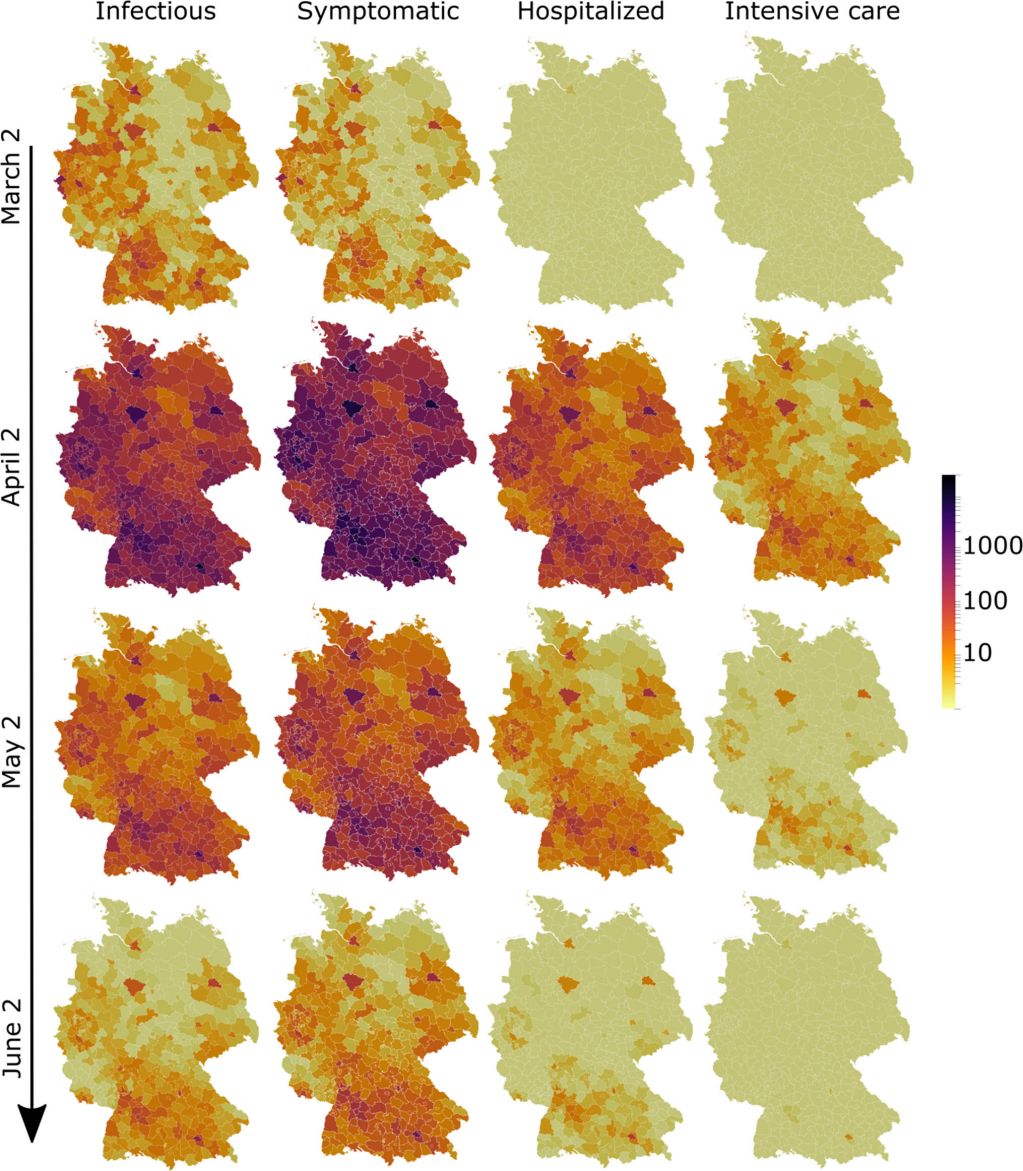
Temporal snapshots of the simulated epidemic spread across Germany at county level from March 2 until June 2 (from top to bottom): people with disease-specific symptoms (left), infectious (middle left), hospitalized (middle right), and patients in intensive care (right)

We note that our fitting procedure only operates on state-based information. To further validate our model, we compare county-wise cumulative infection numbers $\mathbf {Q}$ as reported by the RKI and our model (Fig. 4 left and right, respectively) close to the peak of daily new infections on March 28 and four weeks later on April 25. Judged by the naked eye, the overall agreement for both dates is striking. A spatial correlation of $R^2 = 0.85$ and $R^2 = 0.80$ (both $p < 1e-12$) in March and April, respectively, statistically corroborate the predictive capabilities of our model. Most noticeable deviations are related to individual counties that made the news for especially high infection numbers, such as Rosenheim (Southern Bavaria) or Tirschenreuth (Eastern Bavaria), and positive counter examples such as Hildburghausen (Southern Thuringia).

### Model predictions

Figure 5 shows how the model informs on the temporal evolution of cumulative confirmed cases, with more detailed resolution on the subgroups of symptomatic, infectious, and hospitalized, patients in the ICU, as well as the dead. A first kink in the infectious group is clearly visible at the beginning of March due to the cancellation of major events, which then drops significantly when contact restrictions become effective shortly afterwards.

Figure 6 shows the model predicted spatial distribution at county resolution of infectious, symptomatic, hospitalized, and patients in intensive care, following from the individual disease courses in Fig. 1. We consider a period from early March, where the exponential growth of the disease started in Germany, until early June under the assumption that the contact reduction factors stay in place. In early March, most of the infected were at an early stage of the disease, i.e., most of them were infectious but did not have disease specific symptoms yet (on average, the first symptoms appear on the fifth day after the infection event [26]). This explains the delay in symptomatic infections clearly visible in Fig. 5.

In our model, we assume that most of the symptomatic voluntarily quarantine themselves and no longer infect others, implying that the infectiousness decreases when people move to the symptomatic group (cf. Fig. 1). The infectious state ends at the latest when the symptomatic have been tested positive for the virus and are quarantined. The symptomatic state of Covid-19 lasts approximately nine days on average [26], explaining why the symptomatic group is about double in size compared to the infectious group in Fig. 5.

Figure 6 also shows the delay in Covid-19 cases that need inpatient treatment or even intensive care. As reported in [26], infected are typically hospitalized for nine days after the infection event at a probability of 4.5%. As this is encoded in the courses of disease, the snapshot on March 2 reports hardly any hospitalized patients. According to [26], patients stay in hospital for an average of 14 days, while one fourth of hospitalized patients requires intensive care. Especially the two snapshots in April and May present most of the infected in the symptomatic group, with only a small number requiring inpatient treatment.

**Fig. 7:**
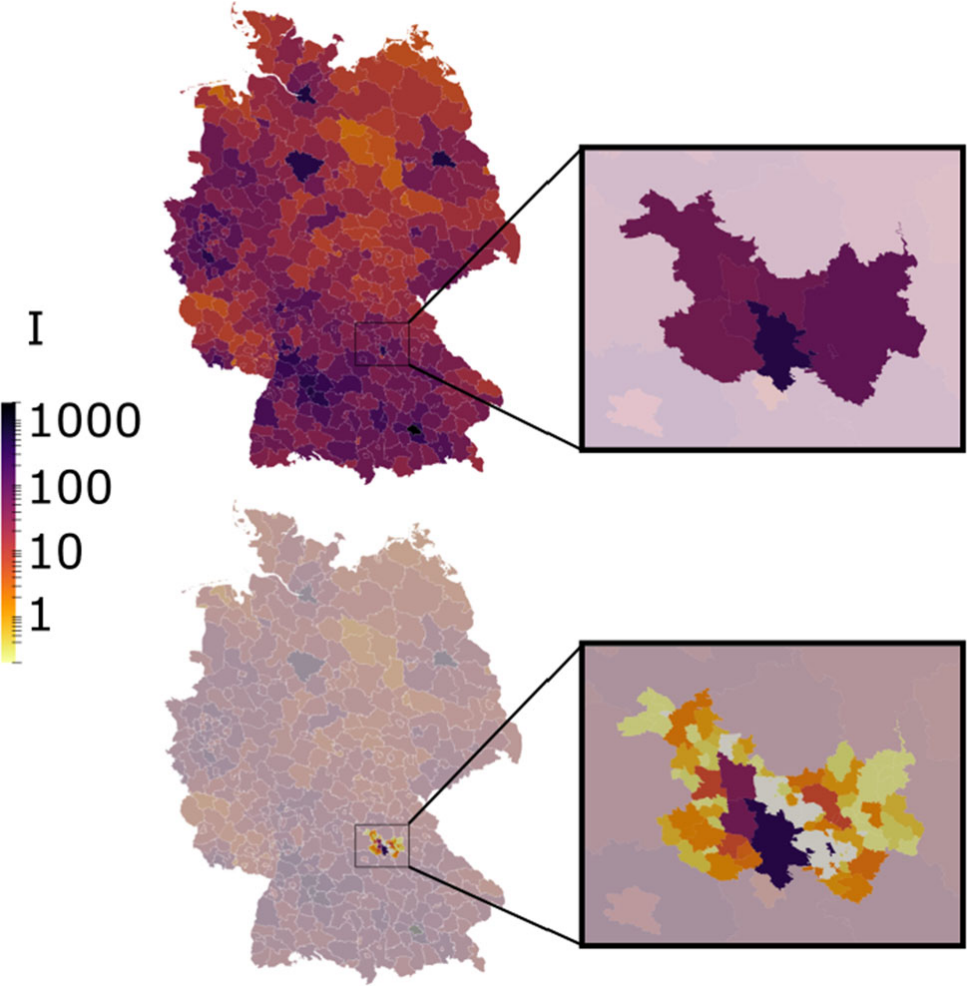
Spatial distribution of the infectious (I) on April 2 at county level for all of Germany (top) and with locally increased resolution to community-level (bottom). The non-densified part of the domain is greyed out for the sake of better visual contrast. Zoomed regions show county- and community resolution for counties Erlangen, Fürth, Nürnberg and their rural surroundings. Note that the proposed macroscopic model reaches its validity limit for very low daily new infections within one subregion

Finally, we show how our model can be adapted to locally increase resolution to individual city- or community level. Figure 7 shows the Germany-wide county-level simulation (top), with a zoom into the metropolitan area of Nürnberg, Erlangen and Fürth and its surrounding counties. Increasing the resolution within this domain to community level (bottom) but maintaining county-level for the rest of Germany leads to a network of 464 nodes. The zoom-in clearly shows that county infections are dominated by their largest cities, following the three purple areas that represent Nürnberg, Fürth and Erlangen from bottom to top, underpinning our formulation of the cross-county infection terms (cf. Eq. (3)). Surrounding communities suffer much less infections due to their much smaller populations. Gray areas correspond to rural public space not assigned to a specific community [27].

## Conclusions and outlook

We have presented a memory-based network model to predict the spatio-temporal outbreak dynamics of the Covid-19 pandemic in Germany. The model considers the effects of political measures, the cancellation of major events and contact restrictions, and the different possible courses of the disease, which is not possible when using traditional SIR-type models. It well represents the evolution of confirmed cases and deaths reported by the RKI from March 2 until April 25. We have then used the model to predict the further developments until June and have provided estimates for the county-wise required capacity of the local health care system, i.e. the number of patients that require hospitalization and even intensive care. Finally, we have demonstrated that the model can be refined to predict the interaction and local outbreak dynamics at community level.

By now, medical data on observed disease progression at most stages during a COVID-19 infection is abundantly available and continues to improve. Our versatile integro-differential approach directly integrates these data into the model and can easily be extended, corroborating its superiority over standard SIR-type models. In general, the model can thus handle an arbitrary number of courses of the disease. Similarly, it may expand to consider region-dependent demographics or varying capacities and quality of treatment of the health-care system.

While the model can serve as a valuable tool to assess the effects of new super spreader events—which may occur any time—on the distribution of cases in Germany, it reaches its validity limit when the number of infections becomes small. To additionally capture this even smaller scale, a coupling to individual agent-based models [15–17] may be beneficial.
